# Efficacy of ceftazidime-avibactam with or without polymyxin for carbapenem-resistant *Klebsiella pneumoniae* infections after initial treatment with polymyxin

**DOI:** 10.1128/spectrum.01770-24

**Published:** 2024-11-21

**Authors:** Jingli Lu, Yani Ma, Zhe Cao, Baoling Zhu, Luna Fan, Haiyang Meng

**Affiliations:** 1 Department of Pharmacy, The First Affiliated Hospital of Zhengzhou University, Zhengzhou, China; 2 Henan Key Laboratory of Precision Clinical Pharmacy, Zhengzhou University, Zhengzhou, China; 3 Department of Pharmacy, The First Affiliated Hospital of Kunming Medical University, Kunming, China; 4 Department of Pharmacy, Zhenping People’s Hospital, Zhenping, China; 5 Department of Pharmacy, Xiangcheng Hospital of Chinese Medicine, Xiangcheng, China; 6 Department of Pharmacy, Huanghe Science and Technology College Affiliated Hospital, Zhengzhou, China; Innovations Therapeutiques et Resistances, Toulouse, France

**Keywords:** ceftazidime-avibactam, polymyxin B, carbapenem-resistant Enterobacteriaceae, salvage therapy

## Abstract

Although polymyxins are a suboptimal option for difficult-to-treat resistant infections, they are still preferred as the first-line treatment, especially in low- and middle-income countries. This study assesses the efficacy of ceftazidime-avibactam (CAZ-AVI) following polymyxin B failure in patients with carbapenem-resistant *Klebsiella pneumoniae* (CRKP) infections. We retrospectively reviewed cases of infections caused by CRKP in adults who received CAZ-AVI as salvage therapy. Clinical features and outcomes were described, and a logistic regression model was used to assess the risk factors associated with in-hospital crude mortality. One hundred and six patients were included in this study. The median age was 56 years. The most common infectious sites were lung. The patients received CAZ-AVI as salvage therapy for a median duration of 9 days following initial treatment with polymyxin B (median, 12.5 days). Also, 91 (85.8%) patients received CAZ-AVI combination therapy, and 34 (32.1%) patients received CAZ-AVI in combination with polymyxin B. The rate of in-hospital crude mortality was 25.5% (27/106), with the highest rate observed in patients treated with regimens containing polymyxin B (41.2%; 14/34). Therapeutic response was observed in 81 (76.4%) patients, with microbiological eradication achieved in 77.1% (74/96) of cases. Multivariable analysis identified that the length of intensive care unit stays, the sequential organ failure assessment (SOFA) score at CAZ-AVI withdrawal, and regimens containing polymyxin B were independently associated with in-hospital mortality, whereas the duration of CAZ-AVI treatment was independently associated with survival. CAZ-AVI salvage therapy demonstrated improved survival outcomes in patients who experienced failure with polymyxin B therapy.

**IMPORTANCE:**

For patients with carbapenem-resistant *Klebsiella pneumoniae* (CRKP) infections, published experience with salvage therapy is limited after the failure of polymyxin-based initial therapy. Here, we found that ceftazidime-avibactam salvage therapy for patients with CRKP infections offers benefit in mortality.

## INTRODUCTION

Infections caused by carbapenem-resistant Enterobacteriaceae (CRE) are an urgent threat to public health (1). Among them, carbapenem-resistant strains of the *Klebsiella pneumoniae* are dominant (2) and associated with high mortality estimated between 33% and 42% (3, 4). Polymyxins (colistin and polymyxin B), which have a rapid bactericidal activity by disruption of the outer membrane integrity of Gram-negative bacteria, are recommended for the treatment of invasive infections due to CRE (5). Especially in low- and middle-income countries where polymyxins are the only accessible therapeutic option, polymyxins are often considered some of the last-resort options for treating CRE infections. Even in countries with the availability of new drugs, clinicians still preferred to use polymyxins against CRE. A previous study has revealed that colistin continued to be used at high rates, even 2 years after the approval of the new β-lactam/β-lactamase inhibitor combination (6). Furthermore, when new antibiotics are approved, the higher patient out-of-pocket costs are prohibitive in clinical practice. In China, polymyxin B became available clinically at the end of 2017, and since then, it has been commonly used in clinical settings, even with the implicit risk of toxicity and relatively poor clinical efficacy (7, 8). Under these circumstances, polymyxins remain an important treatment alternative for carbapenem-resistant infections.

The European Committee on Antimicrobial Susceptibility Testing (EUCAST) and the Clinical and Laboratory Standards Institute (CLSI) have different interpretive criteria to address clinical breakpoints for the polymyxins (9). In 2020, the CLSI removed the susceptible interpretive category for polymyxins, suggesting that no minimum inhibitory concentration (MIC) is able to predict a high probability of therapeutic success for polymyxins (10), whereas the EUCAST maintains susceptible interpretation for colistin (11). These changes were due to the uncertainties about susceptibility testing and the limited clinical utility of these agents. Randomized and observational studies have consistently demonstrated an increased mortality with polymyxins-based therapy for carbapenem-resistant infections compared with the alternative agent ceftazidime-avibactam (CAZ-AVI) (12–14). Polymyxin B treatment failure is encountered in up to 40% of critically ill cases (15). In this regard, after initially being treated with polymyxins, it is important to consider subsequent therapy once clinical failure occurs.

CAZ-AVI is a β-lactam/β-lactamase inhibitor combination approved for the treatment of susceptible Gram-negative infections (16). CAZ-AVI has *in vitro* activity against some carbapenemase-producing bacteria (17). Clinical data have shown that CAZ-AVI achieved satisfactory clinical efficacy in CRE infections (18). Compared with polymyxins, CAZ-AVI decreases the rates of treatment failure and in-hospital mortality (12, 13). Chinese guidelines have endorsed CAZ-AVI as the frontline agent against infections caused by CRE-producing serine carbapenemase, including KPC and OXA-48 (19). Thus, it is important to assess the use of CAZ-AVI after failure of polymyxins-based therapy.

To address this issue, we conducted a retrospective study of 106 patients with carbapenem-resistant *K. pneumoniae* (CRKP) infections, all of whom receive CAZ-AVI as salvage therapy after initially being treated with polymyxin B. Our aim was to describe the clinical features and outcomes of these cases and to specifically explore risk factors of mortality.

## MATERIALS AND METHODS

### Study population

This retrospective observational study was conducted at the First Affiliated Hospital of Zhengzhou University. Patients ≥ 18 years of age who had CRKP infections between June 2020 and May 2023 were eligible for inclusion if the patient (1) had received ≥72 h of polymyxin B as initial therapy (2) and then received ≥72 h of CAZ-AVI with or without polymyxin B as salvage therapy.

### Clinical data collection and definition

All clinical data were recorded in the hospital’s electronic medical record system, which is utilized for all inpatient records at the First Affiliated Hospital of Zhengzhou University. Data extraction was performed systematically by a team of trained researchers. A standardized data extraction form was used to ensure consistency across patient records and facilitate accurate analysis. Detailed data included demographics, comorbidities, severity of illness, microbiological data, laboratory variables, antibiotic treatment, and clinical outcomes. Infection onset was on the day of sampling the index culture. Therapeutic response was defined as partial or complete recovery of signs and symptoms of the infection as documented by the treating physicians at the end of CAZ-AVI treatment. Clinical failure was the persistence or worsening of signs and symptoms of the infection as documented by the treating physicians. Microbiological eradication was defined as a negative culture at the end of CAZ-AVI treatment. We identified patients who received mechanical ventilation, continuous renal replacement therapy (CRRT), and intensive care after infection onset. Hospitalization stay was calculated over the entire admission. The length of intensive care unit (ICU) stay was evaluated from infection to discharge or death. Septic shock was defined as a subset of sepsis, a life-threatening organ dysfunction, with circulatory and cellular/metabolic abnormalities (20).

### Microbiological analysis

Isolate identification and antimicrobial susceptibility testing were performed using the VITEK 2 Compact system (bioMérieux, Marcy l’Etoile, France) and the Phoenix100 automated system (Becton Dickinson, Spark, MD, USA). Susceptibility for colistin, tigecycline, trimethoprim/sulfamethoxazol (TMP/SMX), meropenem, imipenem, aztreonam, amikacin, ciprofloxacin, and levofloxacin was reported. CAZ-AVI susceptibility was not tested during the study period. Results were interpreted in accordance with the CLSI breakpoints (10), except those for colistin, which were interpreted according to the clinical breakpoints published by the EUCAST (11). Phenotypic detection of carbapenemase types was performed with the combined modified carbapenem inactivation method and EDTA-CIM according to the CLSI guidelines (10).

### Statistical analysis

Categorical variables were expressed as frequencies (percentages) and evaluated with the χ² test or two-tailed Fisher’s exact test. Non-normally distributed continuous variables were expressed as the median with interquartile range and evaluated with the Mann–Whitney nonparametric test. For subgroup analysis, we compared patients treated with salvage regimen with or without polymyxin B. Multivariate logistic regression analysis was used to identify independent risk factors for in-hospital mortality and clinical failure. Variables that emerged from univariate analysis with *P* values of < 0.2 or clinical relevance were included in the multivariate model in a stepwise manner. Odds ratios (ORs) and 95% confidence intervals (CIs) were calculated for all associations. All statistical analyses were done with SPSS software (version 25.0). A two-sided *P*-value of <0.05 was considered statistically significant.

## RESULTS

### Baseline characteristics

This study consisted of 106 patients with CRKP infections who received CAZ-AVI as salvage therapy after experiencing clinical failure with polymyxin B. Table 1 shows the characteristics of the patients. The median age was 56 years (43.8–64.5 years), and more than two-thirds were male (71.7%). The most common comorbidities were diabetes and trauma. Lung infections were most common (86/106 [81.1%]), followed by bloodstream infections (43/106 [40.6%]). 85.8% (91/106) were in an ICU at the time of infection. The median length of ICU stay was 22 days (13.8–37.3 days). The majority of patients required mechanical ventilation (92/106 [86.8%]) and CRRT (26/106 [24.5%]). 49.1% (52/106) patients developed septic shock.

**TABLE 1:** Demographic and clinical characteristics of patients treated with ceftazidime-avibactam as salvage therapy^*a*^

Variables	Overall (*N* = 106)	CAZ-AVI-based regimens		*P* value
		Containing polymyxin B (*N* = 34, 32.1%)	Not containing polymyxin B (*N* = 72, 67.9%)	
Age, median (IQR)	56.0 (43.8–64.5)	56.5 (44.5–66.0)	55.5 (42.5–64.0)	0.823
Male	76 (71.7%)	21 (61.8%)	55 (76.4%)	0.119
Underlying condition				
Diabetes	24 (22.6%)	5 (14.7%)	19 (26.4%)	0.180
Trauma	11 (10.4%)	1 (2.9%)	10 (13.9%)	0.166
Coronary heart disease	9 (8.5%)	4 (11.8%)	5 (6.9%)	0.647
Hematological malignancy	6 (5.7%)	2 (5.9%)	4 (5.6%)	1.000
Solid tumors	5 (4.7%)	1 (2.9%)	4 (5.6%)	1.000
Organ transplantation	3 (2.8%)	2 (5.9%)	1 (1.4%)	0.240
Chronic renal insufficiency	2 (1.9%)	2 (5.9%)	0	0.101
Location at the time of infection				
Medical ward	8 (7.5%)	2 (5.9%)	6 (8.3%)	0.325
Surgical ward	7 (6.6%)	4 (11.8%)	3 (4.2%)	
ICU	91 (85.8%)	28 (82.4%)	63 (87.5%)	
Infection sites				
Lung	86 (81.1%)	29 (85.3%)	57 (79.2%)	0.452
Bloodstream	43 (40.6%)	12 (35.3%)	31 (43.1%)	0.447
Intra-abdominal	10 (9.4%)	2 (5.9%)	8 (11.1%)	0.614
Genitourinary	11 (10.4%)	6 (17.6%)	5 (6.9%)	0.179
Skin	1 (0.9%)	1 (2.9%)	0	0.321
Catheter-related	8 (7.5%)	2 (5.9%)	6 (8.3%)	0.959
Intracranial	11 (10.4%)	7 (20.6%)	4 (5.6%)	0.043
Surgical sites	12 (11.3%)	1 (2.9%)	11 (15.3%)	0.123
Number of infection sites				
1	50 (47.2%)	14 (41.2%)	36 (50.0%)	0.653
2	39 (36.8%)	15 (44.1%)	24 (33.3%)	
3	15 (14.2%)	5 (14.7%)	10 (13.9%)	
4	2 (1.9%)	0	2 (2.8%)	
Severity of infection during hospitalization				
ICU admission	98 (92.5%)	32 (94.1%)	66 (91.7%)	0.656
Length of ICU stay (days)	22.0 (13.8–37.3)	20.5 (14.8–41.8)	22.0 (12.2–36.8)	0.781
Hospitalization stays (days)	43.0 (30.5–73.5)	50.0 (28.5–76.0)	42.5 (31.3–71.5)	0.903
Mechanical ventilation	92 (86.8%)	33 (97.1%)	59 (81.9%)	0.066
CRRT	26 (24.5%)	14 (41.2%)	12 (16.7%)	0.006
Septic shock	52 (49.1%)	20 (58.8%)	32 (44.4%)	0.167
SOFA score at CAZ-AVI initiation	5.5 (3.0–8.0)	7.0 (3.0–9.3)	5.0 (3.0–7.8)	0.111
SOFA score at CAZ-AVI withdrawal	4.0 (2.0–8.0)	7.0 (3.0–9.5)	4.0 (2.0–6.8)	0.005
Surgical debridement	42 (39.6%)	15 (44.1%)	27 (37.5%)	0.516
Carbapenemases				
MBL	1 (0.9%)	0	1 (1.4%)	0.898
SBL	57 (53.8%)	18 (52.9%)	39 (54.2%)	
MBL + SBL	2 (1.9%)	0	2 (2.8%)	
Not tested	46 (43.4%)	16 (47.1%)	30 (41.7%)	

^*a*^ CAZ-AVI, ceftazidime-avibactam; CRKP, carbapenem-resistant *Klebsiella pneumoniae*; CRRT, continuous renal replacement therapy; ICU, intensive care unit; MBL, metallo-β-lactamases; SBL, serine β-lactamases; SOFA, sequential organ failure assessment.

Thirty-four (32.1%) patients received CAZ-AVI-based regimens containing polymyxin B. Intracranial infections receiving CRRT occurred more often in patients who were treated with antibiotic regimens containing polymyxin B. At the start of CAZ-AVI treatment, SOFA score was similar between the two groups. After the completion of CAZ-AVI treatment, SOFA score was significantly higher in patients treated with antibiotic regimens containing polymyxin B than those not containing polymyxin B (Table 1).

### Microbiological characteristics

Table 2 shows the resistance patterns of the 103 isolates. All tested 103 isolates were resistant to meropenem and imipenem, with minimum inhibitory concentrations of ≥16 mg/L (Table 2). The majority of isolates were resistant to aztreonam (100 [98.0%] of 102 tested isolates), ciprofloxacin (99 [96.1%] of 103 tested isolates), levofloxacin (98 [95.1%] of 103 tested isolates), amikacin (84 [81.6%] of 103 tested isolates), and TMP/SMX (65 [64.4%] of 101 tested isolates). 13.6% (14 of 103 tested isolates) were resistant to colistin, and 7.8% (8 of 102 tested isolates) were resistant to tigecycline. Of the 60 isolates tested for carbapenemase production, 1 produced metallo-β-lactamases (MBL), 57 produced serine β-lactamases (SBL), and 2 produced both SBL and MBL (Table 1).

**TABLE 2:** Antimicrobial Susceptibility Testing for CRKP Isolates^*a*^

Antibiotic	Isolates tested	Sensitive	Intermediate	Resistance
Colistin	103	89 (86.4%)	0	14 (13.6%)
Tigecycline	102	83 (81.4%)	11 (10.8%)	8 (7.8%)
TMP/SMX	101	36 (35.6%)	0	65 (64.4%)
Meropenem	103	0	0	103 (100.0%)
Imipenem	103	0	0	103 (100.0%)
Aztreonam	102	2 (2.0%)	0	100 (98.0%)
Amikacin	103	19 (18.4%)	0	84 (81.6%)
Ciprofloxacin	103	4 (3.9%)	0	99 (96.1%)
Levofloxacin	103	3 (2.9%)	2 (1.9%)	98 (95.1%)

^*a*^ CRKP, carbapenem-resistant *Klebsiella pneumoniae*; TMP/SMX, trimethoprim/sulfamethoxazole.

### Treatment

Before CAZ-AVI treatment, all 106 patients received polymyxin B for a median duration of 12.5 days (7–21 days), and the median time from the onset of infection to the start of polymyxin B was 2 days (–1 to 5 days). During treatment with polymyxin B, 23 isolates exhibited resistance to polymyxin B. Thirty-eight patients had plasma concentration of polymyxin B, with a median AUC_SS, 24h_ of 63.8 mg·h/L. Among them, 31.6% (12/38) did not achieve the AUC_SS, 24h_ target of >50 mg·h/L as recommended by the guidelines (Table 3).

**TABLE 3:** Treatment and outcomes^*a*^

Characteristic	Overall (*N* = 106)	CAZ-AVI-based regimens		*P* value
		Containing polymyxin B (*N* = 34, 32.1%)	Not containing polymyxin B (*N* = 72, 67.9%)	
Polymyxin B prior to CAZ-AVI				
Infection days before polymyxin B	2 (-1.0–5.0)	2.0 (-5.5–5.3)	2.0 (-0.8–5.0)	0.696
Days of polymyxin B treatment	12.5 (7.0–21.0)	17.5 (14.0–26.3)	10.0 (6.0–17.8)	0.000
Polymyxins resistance	23 (21.7%)	6 (17.6%)	17 (23.6%)	0.487
Polymyxin B plasma concentration				
Estimated AUC_SS, 24h_ (mg·h/L)^*b*^	63.8 (42.6–87.0)	64.5 (35.8–95.7)	64.5 (35.8–95.7)	0.856
Estimated AUC_SS, 24h_ < 50 mg·h/L	12/38 (31.6%)	6/14 (42.9%)	6/24 (25.0%)	0.296
Salvage treatment due to				
Treatment failure	97 (91.5%)	32 (94.1%)	65 (90.3%)	0.847
Nephrotoxicity	3 (2.8%)	1 (2.9%)	2 (2.8%)	
Infection relapse	6 (5.7%)	1 (2.9%)	5 (6.9%)	
CAZ-AVI treatment				
Monotherapy	15 (14.2%)	0	15 (20.8%)	0.010
Combination therapy	91 (85.8%)	34 (100.0%)	57 (79.2%)	
Infection days before CAZ-AVI	11.0 (6.0–23.0)	7.5 (4.8–13.3)	14.0 (7.0–24.8)	0.024
Days of CAZ-AVI treatment	9.0 (6.0–14.3)	13.0 (6.0–16.5)	9.0 (6.0–13.0)	0.060
CAZ-AVI combination containing				
Polymyxin B	34 (32.1%)	34 (100%)	0	/
Carbapenems	38 (35.8%)	11 (32.4%)	27 (37.5%)	0.606
Tigecycline	21 (19.8%)	8 (23.5%)	13 (18.1%)	0.509
Aztreonam	31 (29.2%)	5 (14.7%)	26 (36.1%)	0.024
Other β-lactams	8 (7.5%)	3 (8.8%)	5 (6.9%)	1.000
Clinical outcomes				
Therapeutic response	81 (76.4%)	22 (64.7%)	59 (81.9%)	0.051
Clinical failure	25 (23.6%)	12 (35.3%)	13 (18.1%)	
Microbiologic eradication	74/96 (77.1%)	26/32 (81.3%)	48/64 (75.0%)	0.492
In-hospital crude mortality	27 (25.5%)	14 (41.2%)	13 (18.1%)	0.011

^*a*^ CAZ-AVI, ceftazidime-avibactam.

^*b*^ 38 patients were detected for polymyxin B concentration, 14 cases in group with polymyxin B, 24 in group without polymyxin B.

After the initial treatment with polymyxin B, 97 (91.5%) patients were considered treatment failure, 3 (2.8%) patients developed nephrotoxicity, and 6 (5.7%) patients had infection relapse. All 106 patients were treated with CAZ-AVI for a median duration of 9 days (6.0–14.3 days), and the median time from the onset of infection to the initiation of CAZ-AVI was 11 days (6–23 days). Of the 106 patients, 15 (14.2%) received CAZ-AVI monotherapy and 91 (85.8%) received CAZ-AVI combination therapy. Then, 38 (35.8%) patients received an antibiotic regimen containing carbapenem, 31 (29.2%) aztreonam, 21 (19.8%) tigecycline, and 8 (7.5%) other β-lactams. Thirty-seven patients received combination antibiotic therapy with at least three different classes of drugs. The antibiotic treatment regimens stratified by infection sites are provided in Table S1.

### Outcomes

For all patients, the rate of in-hospital crude mortality was 25.5% (27/106) (Table 3). Regarding the infection sites, the highest rate of mortality was observed in patients with intracranial infection (36.4% [4/11]), followed by intra-abdominal infection (30.0% [3/10]), bloodstream infection (25.6% [11/43]), and pneumonia (25.6% [22/86]) (Table S2). For the subgroups, the mortality rate was 41.2% (14/34) for patients treated with regimens containing polymyxin B, and 33.3% (7/21) for tigecycline, 25.8% (8/31) for aztreonam, and 18.4% (7/38) for carbapenems (Table S3).

Therapeutic response was observed in 81 (76.4%) patients (Table 3). The rate of therapeutic response was 76.7% (66/86) for pneumonia, 74.4% (32/43) for bloodstream infection, and 91.7% (11/12) for infection in surgical sites (Table S2). Patients treated with regimens containing tigecycline had the lowest rate of therapeutic response (52.4% [11/21]), whereas those treated with regimens containing carbapenems had the rate of 86.8% (33/38) (Table S3).

A total of 96 patients had follow-up microbiological data, of which 74 (77.1%) had microbiological eradication (Table 3). In the subgroup analysis, microbiological eradication was achieved in 75.9% (60/79) of cases with pneumonia and 87.2% (34/39) of those with bloodstream infections (Table S3). Microbiological eradication rates were 64.3% (18/28) and 82.9% (29/35) for patients treated with regimens containing aztreonam and carbapenems, respectively (Table S3).

### Risk factors for in-hospital mortality and clinical failure

The results of the univariate analyses of risk factors for in-hospital mortality and clinical failure are shown in Tables S4 and S5, respectively. After adjusting for confounding in the multivariable analysis, the length of ICU stays (OR 1.051, 95% CI 1.015–1.088), SOFA score at CAZ-AVI withdrawal (OR 1.172, 95% CI 1.024–1.341), and combination containing polymyxin B (OR 3.507, 95% CI 1.047–11.748) were independent factors of in-hospital mortality, whereas the duration of CAZ-AVI treatment (OR 0.897, 95% CI 0.808–0.997) was independently associated with survival (Table 4).

**TABLE 4:** Logistic regression analysis for risk factors associated with in-hospital mortality^*a*^

Risk factor	Non-survival (*N* = 27)	Survival (*N* = 79)	Multivariate Analyses	
			OR (95% CI)	*P* value
Age	57 (48–69)	55 (40–64)	1.022 (0.979–1.065)	0.321
Male	22 (81.5%)	54 (68.4%)	2.198 (0.539–8.960)	0.272
Length of ICU stays	22 (16–47)	22 (12–35)	1.051 (1.015–1.088)	0.005
SOFA score at CAZ-AVI withdrawal	8.0 (4.0–11.0)	4.0 (2.0–7.0)	1.172 (1.024–1.341)	0.021
Days of CAZ-AVI treatment	9 (4-13)	10 (7–15)	0.897 (0.808–0.997)	0.044
CAZ-AVI combination containing carbapenems	7 (25.9%)	31 (39.2%)	0.576 (0.181–1.836)	0.351
CAZ-AVI combination containing polymyxin B	14 (51.9%)	20 (25.3%)	3.507 (1.047–11.748)	0.042

^*a*^ CAZ-AVI, ceftazidime-avibactam; ICU, intensive care unit; SOFA, sequential organ failure assessment.

Intracranial infection (OR 13.590, 95% CI 1.982–93.180), SOFA score at CAZ-AVI withdrawal (OR 1.251, 95% CI 1.059–1.478), and combination containing tigecycline (OR 4.619, 95% CI 1.245–17.134) were independent predictors of clinical failure, whereas combination containing carbapenems (OR 0.219, 95% CI 0.053–0.906) was the protective factor (Table 5).

**TABLE 5:** Logistic regression analysis for risk factors associated with clinical failure^*a*^

Risk factor	Clinical failure (*N* = 25)	Therapeutic response (*N* = 81)	Multivariate analyses	
			OR (95% CI)	*P* value
Genitourinary infection	6 (24.0%)	5 (6.2%)	6.988 (0.994–49.129)	0.051
Surgical site infection	1 (4.0%)	11 (13.6%)	0.201 (0.015–2.743)	0.201
Intracranial infection	5 (20.0%)	6 (7.4%)	13.590 (1.982–93.180)	0.008
Number of infection sites	2 (1-3)	2 (1-2)	1.247 (0.541–2.879)	0.604
CRRT	9 (36.0%)	17 (21.0%)	1.663 (0.356–7.764)	0.518
SOFA score at CAZ-AVI withdrawal	7.0 (4.0–11.5)	4.0 (2.0–7.0)	1.251 (1.059–1.478)	0.008
CAZ-AVI combination containing tigecycline	10 (40.0%)	11 (13.6%)	4.619 (1.245–17.134)	0.022
CAZ-AVI combination containing carbapenems	5 (20.0%)	33 (40.7%)	0.219 (0.053–0.906)	0.036
CAZ-AVI combination containing polymyxin B	12 (48.0%)	22 (27.2%)	0.598 (0.155–2.305)	0.455

^*a*^ CAZ-AVI, ceftazidime-avibactam; CRRT, continuous renal replacement therapy; SOFA, sequential organ failure assessmen.

## DISCUSSION

Although a few studies on the use of polymyxins have demonstrated that these antibiotics have increased mortality compared with alternative agents (12–14), they are still widely used as the first-line treatment, especially in low-income countries. However, after the failure of polymyxins-based initial therapy, published experience with salvage therapy is limited. Our study focused on the utilization of CAZ-AVI after polymyxin failure, which provide significant insights into the treatment options for patients requiring salvage therapy.

In our study, the most frequent reason for the switch to CAZ-AVI was treatment failure and disease progression with polymyxin B, accounting for 90% of patients. In addition, nephrotoxicity following polymyxin B occurred and led to discontinuation in 2.8% of cases. 5.7% of patients had recurrent infections. Two reasons were postulated for polymyxin B failure. It is possible that the current dosage regimens for some cases were insufficient to drive protection (21, 22) because polymyxin plasma concentrations are suboptimal in 31.6% of 38 patients, with an estimated AUC_SS, 24h_ < 50 mg·h/L. In addition to poor clinical outcomes, exposure to suboptimal polymyxin B concentrations may increase the likelihood of antibiotic resistance, especially against polymyxin-heteroresistant strains that have been detected in CRKP clinical isolates (23–25). In the current study, we did not detect heteroresistance, but we found that nine polymyxin-susceptible *K. pneumoniae* isolates developed resistance during polymyxin B treatment. Due to the collective amount of evidence regarding the emergence of heteroresistance to polymyxin, we cannot rule out the possibility of the presence of heteroresistance in our cohort, which may result in the failure of antibiotic treatment for infections caused by bacteria that are classified as antibiotic susceptible.

Delays in effective antibiotic therapy have been associated with increased mortality (26–28). It is reasonable to expect worse outcomes in patients with salvage therapy. Our CAZ-AVI salvage therapy produced a therapeutic response in 76.4% of patients, with microbiological eradication of 77.1% in the follow-up isolate culture. The in-hospital mortality rate of 25.5% is unexpectedly low. Several retrospective studies have reported that the 30-day mortality rates with CAZ-AVI-based regimens reached 30% in critically ill patients with CRE infections (29, 30). In comparison, our study excluded patients who were treated with CAZ-AVI for less than 72 h, which may have excluded critically ill patients who could not receive an adequate course of CAZ-AVI treatment (30). When compared with our study, one study included more patients who had a comorbidity of organ transplantation, which was an independently negative factor for 30-day survival (29). These may account for the observed decrease in mortality rate compared with the previous studies.

The mortality rate is also lower than those from published studies on the use of CAZ-AVI as salvage therapy (31–34). The results of 36 case series on infections caused by carbapenem-resistant organisms showed a 73.7% clinical and/or microbiological cure, with a mortality rate of 41.7% in patients with CAZ-AVI salvage therapy (31). Other clinical experiences resulted in an all-cause mortality of 42.9% in 21 liver transplantation recipients with CRKP infections (32), and 51.7% in 29 patients with infections caused by *Enterobacteriales* co-resistant to carbapenems and polymyxins (34). These three studies may have observed higher mortality due to different bacterial pathogens, different patient populations, different lengths of time for recording mortality, and an overall smaller sample size. There is a retrospective study from Italy that included 138 cases, reporting a 30-day mortality of 34.1% and CAZ-AVI as the sole independent predictor of survival (33), even though a longer delay in starting CAZ-AVI in our study (median, 7 days vs 11 days from infection onset). The disparity with earlier reports may reflect the difference in antibiotic regimens prior to CAZ-AVI salvage therapy because half of the patients received carbapenems, tigecycline, or fosfomycin as initial therapy in the Italian cohort. It is also possible that the difference in the proportion of bloodstream infections in the present study, compared with prior studies (40.6% vs 75.4%) may account for the discrepancies (33).

Despite the mortality benefit of CAZ-AVI in difficult-to-treat resistant infections, suitable combined agents with CAZ-AVI remain unclear (35). Interestingly, data from the current study suggest that the addition of polymyxin B to CAZ-AVI worsened the outcomes in these patients, with the highest mortality rate of 41.2%. *In vitro* findings have supported that CAZ-AVI plus polymyxins did not have synergic activity against CRE (36–38). Although the combined use with polymyxins might not have altered the efficacy of CAZ-AVI, another factor that likely contributed to the high rates of mortality may be the accumulated toxicity from polymyxin-based therapy. As demonstrated by the laboratory parameters in evaluating kidney function, a significant decline in serum creatinine was found after CAZ-AVI was substituted for polymyxin B. By contrast, serum creatinine increased by 18.5 umol/L in patients treated with CAZ-AVI in combination with polymyxin B (Table S6).

Similarly, CAZ-AVI salvage therapy containing tigecycline was associated with clinical failure. This result is opposed to previous findings where tigecycline was a preferred combination to improve clinical outcomes for patients with CRKP infections (29, 39). Compared with these two studies, at present, the major sites for infections were the lung and bloodstream, settings where tigecycline is not a first choice owing to its distribution profile (40). Although the underlying reason for the observed discrepancy remains to be determined, tigecycline-induced liver injury should be considered. Accumulating data have revealed that tigecycline is associated with liver injury (41, 42). In support of this, an improvement in liver function was seen without tigecycline, as demonstrated by a significant decrease in total bilirubin and increase in albumin during salvage treatment, which did not occur with tigecycline combination therapy (Table S7). Findings of a multicenter retrospective study presented a 10.3% tigecycline-induced liver injury, and tigecycline-associated risk became stronger in patients with abnormal baseline liver enzyme (41). Importantly, CAZ-AVI-associated increases in liver enzymes have been reported in previous studies (29). As such, the combination of CAZ-AVI and tigecycline may increase the risk of liver injury. However, the analysis of 105 patients from our study showed that higher levels of total bilirubin were not associated with in-hospital mortality.

An important finding from our analysis was that carbapenem was an independent protective factor for clinical failure during CAZ-AVI salvage treatment. In line with our results, previous studies have confirmed that CAZ-AVI combined with carbapenem was related to the increase in microbiological eradication (43) and the decrease in 30-day mortality rates (39). This observation was further underpinned by *in vitro* studies that showed the synergistic activity in combination of CAZ-AVI with carbapenems against all KPC-Kp isolates (44).

We acknowledge several limitations of our study. We used retrospective data that did not fully record clinical variables. Additionally, we did not conduct a case–control matching to compare with salvage therapy regimens that did not include CAZ-AVI. Lastly, we were unable to fully characterize the reason for treatment failure of polymyxin B-based therapy as not all patients detected polymyxin plasma concentration and no patient was tested for heteroresistance to polymyxin.

In conclusion, CAZ-AVI salvage therapy has benefits on in-hospital mortality in patients with polymyxin-based initial therapy. However, an increase in mortality rate is seen for combination therapy with polymyxin B, regardless of the disease severity and duration of CAZ-AVI.

## SUPPLEMENTAL MATERIAL

Supplemental tables spectrum.01770-24-s0001.docx Tables S1 to S7.

ASM does not own the copyrights to Supplemental Material that may be linked to, or accessed through, an article. The authors have granted ASM a non-exclusive, world-wide license to publish the Supplemental Material files. Please contact the corresponding author directly for reuse.
