# Supplementary material for: Efficacy of ceftazidime-avibactam with or without polymyxin for carbapenem-resistant Klebsiella pneumoniae infections after initial treatment with polymyxin
Source: Microbiol Spectr. 2024 Nov 21;13(1):e01770-24. doi: 10.1128/spectrum.01770-24 (PMC11705916; doi:10.1128/spectrum.01770-24)
Supplement: Supplemental tables — Tables S1 to S7. [file spectrum.01770-24-s0001.docx]

**Supplementary Table 1 Antibiotic treatment regimens by infection sites**

| **Antimicrobial regimen** | **Lung** | **Bloodstream** | **Intra-abdominal** | **Genitourinary** | **Skin** | **Catheter-related** | **Intracranial** | **Surgical sites** |
| --- | --- | --- | --- | --- | --- | --- | --- | --- |
| **CAZ-AVI monotherapy** | 9 | 7 | 2 | 0 | 0 | 0 | 0 | 4 |
| **CAZ-AVI combination therapy** |  |  |  |  |  |  |  |  |
| **2 antibiotics combination** |  |  |  |  |  |  |  |  |
| CAZ-AVI + aztreonam | 14 | 4 | 1 | 1 | 0 | 2 | 1 | 1 |
| CAZ-AVI + polymyxin B | 11 | 1 | 0 | 1 | 0 | 1 | 3 | 1 |
| CAZ-AVI + carbapenems | 14 | 10 | 1 | 1 | 0 | 2 | 3 | 2 |
| CAZ-AVI + tigecycline | 6 | 3 | 1 | 0 | 0 | 1 | 0 | 1 |
| **≥ 3 antibiotics combination** |  |  |  |  |  |  |  |  |
| CAZ-AVI + aztreonam + polymyxin B | 1 | 2 | 1 | 0 | 0 | 0 | 0 | 0 |
| CAZ-AVI + other β-lactams + polymyxin B | 3 | 1 | 0 | 1 | 0 | 0 | 0 | 0 |
| CAZ-AVI + carbapenems + aztreonam | 5 | 2 | 0 | 0 | 0 | 0 | 0 | 0 |
| CAZ-AVI + carbapenems + polymyxin B | 5 | 3 | 0 | 2 | 0 | 1 | 2 | 0 |
| CAZ-AVI + carbapenems + other β-lactams | 3 | 1 | 0 | 0 | 0 | 0 | 0 | 1 |
| CAZ-AVI + tigecycline + aztreonam | 3 | 0 | 3 | 1 | 0 | 1 | 0 | 2 |
| CAZ-AVI + tigecycline + polymyxin B | 5 | 2 | 1 | 1 | 1 | 0 | 1 | 0 |
| CAZ-AVI + tigecycline + other β-lactams | 1 | 0 | 0 | 0 | 0 | 0 | 0 | 0 |
| CAZ-AVI + carbapenems + tigecycline + aztreonam | 0 | 2 | 0 | 0 | 0 | 0 | 0 | 0 |
| CAZ-AVI + carbapenems + tigecycline + polymyxin B | 1 | 1 | 0 | 0 | 0 | 0 | 1 | 0 |
| CAZ-AVI + carbapenems + aztreonam + polymyxin B | 3 | 2 | 0 | 1 | 0 | 0 | 0 | 0 |

Abbreviations: CAZ-AVI, ceftazidime-avibactam.

**Supplementary Table 2 Outcomes in patients with CRKP infections by infection sites**

| **Characteristic** | **In-hospital**  **crude mortality** | **Microbiologic eradication** | **Clinical outcomes** | | |
| --- | --- | --- | --- | --- | --- |
|  |  |  |  | **Therapeutic response** | **Clinical failure** |
| **Lung, n=86** | 22 (25.6%) | 60/79 (75.9%) |  | 66 (76.7%) | 20 (23.3%) |
| **Bloodstream, n=43** | 11 (25.6%) | 34/39 (87.2%) |  | 32 (74.4%) | 11 (25.6%) |
| **Intra-abdominal, n=10** | 3 (30.0%) | 5/9 (55.6%) |  | 8 (80.0%) | 2 (20.0%) |
| **Genitourinary, n=11** | 3 (27.3%) | 7/11 (63.6%) |  | 5 (45.5%) | 6 (54.5%) |
| **Skin, n=1** | 0 | 1/1 (100.0%) |  | 0 | 1 (100.0%) |
| **Catheter-related, n=8** | 2 (25.0%) | 6/8 (75.0%) |  | 6 (75.0%) | 2 (25.0%) |
| **Intracranial, n=11** | 4 (36.4%) | 8/11 (72.7%) |  | 6 (54.5%) | 5 (45.5%) |
| **Surgical sites, n=12** | 2 (16.7%) | 8/11 (72.7%) |  | 11 (91.7%) | 1 (8.3%) |

Abbreviations: CRKP, carbapenem-resistant *Klebsiella pneumoniae*.

**Supplementary Table 3 Outcomes in patients with CRKP infections by treatment strategy**

| **Characteristic** | **In-hospital**  **crude mortality** | **Microbiologic eradication** | **Clinical outcomes** | | |
| --- | --- | --- | --- | --- | --- |
|  |  |  |  | **Therapeutic response** | **Clinical failure** |
| **Monotherapy, n=15** | 4 (26.7%) | 10/13 (76.9%) |  | 13 (86.7%) | 2 (13.3%) |
| **Combination therapy, n=91** | 23 (25.3%) | 64/83 (77.1%) |  | 68 (74.7%) | 23 (25.3%) |
| **Combination containing polymyxin B, n=34** | 14 (41.2%) | 26/32 (81.2%) |  | 22 (64.7%) | 12 (35.3%) |
| **Combination containing carbapenems, n=38** | 7 (18.4%) | 29/35 (82.9%) |  | 33 (86.8%) | 5 (13.2%) |
| **Combination containing tigecycline, n=21** | 7 (33.3%) | 14/18 (77.8%) |  | 11 (52.4%) | 10 (47.6%) |
| **Combination containing aztreonam, n=31** | 8 (25.8%) | 18/28 (64.3%) |  | 26 (83.9%) | 5 (16.1%) |
| **Combination containing other β-lactams, n=8** | 1 (12.5%) | 5/6 (83.3%) |  | 7 (87.5%) | 1 (12.5%) |

Abbreviations: CRKP, carbapenem-resistant *Klebsiella pneumoniae*.

**Supplementary Table 4 Univariate analyses for risk factors of morality**

| **Risk factor** | **OR (95% CI)** | ***P*** |
| --- | --- | --- |
| **Male** | 2.037 (0.691-6.002) | 0.197 |
| **Older age** | 1.023 (0.990-1.057) | 0.178 |
| **Underlying condition** |  |  |
| Diabetes | 0.513 (0.158-1.664) | 0.226 |
| Trauma | 0.265 (0.032-2.177) | 0.217 |
| Coronary heart disease | 1.521 (0.353-6.553) | 0.574 |
| Hematological malignancy | 0.569 (0.064-5.102) | 0.615 |
| Solid tumors | 2.027 (0.320-12.830) | 0.453 |
| Organ transplantation | 1.481 (0.129-17.011) | 0.753 |
| **Infection sites** |  |  |
| Lung | 1.031 (0.336-3.167) | 0.957 |
| Bloodstream | 1.010 (0.415-2.458) | 0.983 |
| Intra-abdominal | 1.286 (0.308-5.368) | 0.730 |
| Genitourinary | 1.109 (0.272-4.523) | 0.885 |
| Skin | / |  |
| Catheter-related | 0.973 (0.184-5.137) | 0.975 |
| Intracranial | 1.789 (0.480-6.664) | 0.386 |
| Surgical sites | 0.552 (0.113-2.695) | 0.463 |
| **Number of infection sites** | 1.076 (0.616-1.878) | 0.797 |
| **Severity of infection** |  |  |
| Length of ICU stay (days) | 1.023 (0.999-1.048) | 0.064 |
| Mechanical ventilation | 0.833 (0.238-2.914) | 0.775 |
| CRRT | 1.427 (0.536-3.799) | 0.477 |
| Septic shock | 0.952 (0.397-2.283) | 0.913 |
| SOFA score at CAZ-AVI initiation | 1.077 (0.959-1.210) | 0.209 |
| SOFA score at CAZ-AVI withdrawal | 1.232 (1.093-1.388) | 0.001 |
| **Surgical debridement** | 1.065 (0.437-2.594) | 0.891 |
| **PMB treatment prior to CAZ-AVI** |  |  |
| Infection days before polymyxin B | 0.977 (0.929-1.027) | 0.356 |
| Days of polymyxin B treatment | 1.018 (0.977-1.060) | 0.403 |
| **Combination therapy** | 0.930 (0.270-3.208) | 0.909 |
| **Infection days before CAZ-AVI** | 0.991 (0.958-1.025) | 0.603 |
| **Days of CAZ-AVI treatment** | 0.957 (0.895-1.022) | 0.190 |
| **CAZ-AVI combination containing** |  |  |
| Polymyxin B | 3.177 (1.280-7.888) | 0.013 |
| Carbapenems | 0.542 (0.205-1.432) | 0.217 |
| Tigecycline | 1.625 (0.576-4.581) | 0.359 |
| Aztreonam | 1.025 (0.393-2.672) | 0.959 |
| Other β-lactams | 0.396 (0.046-3.372) | 0.396 |

Abbreviations: CAZ-AVI, ceftazidime-avibactam; CRKP, carbapenem-resistant *Klebsiella pneumoniae*; CRRT, continuous renal replacement therapy; ICU, intensive care unit; SOFA, sequential organ failure assessment.

**Supplementary Table 5 Univariate analyses for risk factors of clinical failure**

| **Risk factor** | **OR (95% CI)** | ***P*** |
| --- | --- | --- |
| **Male** | 1.786 (0.602-5.298) | 0.296 |
| **Older age** | 1.010 (0.978-1.043) | 0.554 |
| **Underlying condition** |  |  |
| Diabetes | 0.816 (0.270-2.467) | 0.718 |
| Trauma |  |  |
| Coronary heart disease | 0.919 (0.178-4.737) | 0.920 |
| Hematological malignancy | 0.633 (0.070-5.690) | 0.683 |
| Solid tumors | 0.802 (0.085-7.524) | 0.847 |
| Organ transplantation | 1.646 (0.143-18.950) | 0.689 |
| **Infection sites** |  |  |
| Lung | 0.909 (0.294-2.811) | 0.869 |
| Bloodstream | 1.203 (0.486-2.979) | 0.689 |
| Intra-abdominal | 0.793 (0.157-4.005) | 0.779 |
| Genitourinary | 4.800 (1.323-17.418) | 0.017 |
| Skin | / |  |
| Catheter-related | 1.087 (0.205-5.757) | 0.922 |
| Intracranial | 3.125 (0.864-11.298) | 0.082 |
| Surgical sites | 0.265 (0.033-2.163) | 0.215 |
| **Number of infection sites** | 1.553 (0.888-2.718) | 0.123 |
| **Severity of infection** |  |  |
| Length of ICU stay (days) | 0.997 (0.972-1.023) | 0.818 |
| Mechanical ventilation | 0.739 (0.210-2.601) | 0.638 |
| CRRT | 2.118 (0.798-5.621) | 0.132 |
| Septic shock | 0.767 (0.311-1.889) | 0.563 |
| SOFA score at CAZ-AVI initiation | 1.149 (1.017-1.298) | 0.026 |
| SOFA score at CAZ-AVI withdrawal | 1.218 (1.082-1.372) | 0.001 |
| **Surgical debridement** | 1.569 (0.635-3.879) | 0.329 |
| **PMB treatment prior to CAZ-AVI** |  |  |
| Infection days before polymyxin B | 0.969 (0.919-1.021) | 0.235 |
| Days of polymyxin B treatment | 1.021 (0.979-1.064) | 0.337 |
| **Combination therapy** | 2.199 (0.461-10.483) | 0.323 |
| **Infection days before CAZ-AVI** | 0.985 (0.950-1.022) | 0.422 |
| **Days of CAZ-AVI treatment** | 0.968 (0.907-1.033) | 0.322 |
| **CAZ-AVI combination containing** |  |  |
| Polymyxin B | 2.476 (0.982-6.242) | 0.055 |
| Carbapenems | 0.364 (0.124-1.066) | 0.065 |
| Tigecycline | 4.242 (1.527-11.788) | 0.006 |
| Aztreonam | 0.529 (0.179-1.566) | 0.250 |
| Other β-lactams | 0.440 (0.052-3.764) | 0.454 |

Abbreviations: CAZ-AVI, ceftazidime-avibactam; CRKP, carbapenem-resistant *Klebsiella pneumoniae*; CRRT, continuous renal replacement therapy; ICU, intensive care unit; SOFA, sequential organ failure assessment.

**Supplementary Table 6 Comparison of safety between CAZ-AVI treatment** **containing and not containing polymyxin B**

| **Treatment strategy** | **Laboratory variables** | **Before salvage treatment** | **After salvage treatment** | ***P*** |
| --- | --- | --- | --- | --- |
| **CAZ-AVI combination**  **not containing polymyxin B** | Creatinine (umol/L), n=70 | 68.5 (47.0-127.3) | 51.0 (34.8-92.8) | 0.026 |
|  | eGFR, n=70 | 96.5 (57.9-124.7) | 114.7 (75.9-137.6) | 0.032 |
|  | ALT (U/L), n=70 | 29.0 (14.0-58.8) | 36.5 (7.8-61.8) | 0.528 |
|  | AST (U/L), n=70 | 36.0 (23.0-61.0) | 37.0 (22.0-63.3) | 0.622 |
|  | Total bilirubin (umol/L), n=70 | 13.6 (7.2-26.9) | 9.4 (5.8-18.2) | 0.058 |
|  | Albumin (g/L), n=70 | 28.3 (26.2-32.7) | 32.3 (29.2-34.6) | 0.000 |
| **CAZ-AVI combination**  **containing polymyxin B** | Creatinine (umol/L), n=34 | 67.0 (33-151.3) | 85.5 (32.2-179.5) | 0.659 |
|  | eGFR, n=33 | 98.5 (33.8-127.0) | 82.5 (31.1-121.7) | 0.686 |
|  | ALT (U/L), n=34 | 33.5 (15.5-57.5) | 31.5 (13.0-54.3) | 0.854 |
|  | AST (U/L), n=34 | 42.0 (22.8-58.0) | 37.5 (22.7-72.0) | 0.859 |
|  | Total bilirubin (umol/L), n=34 | 19.0 (9.8-33.1) | 9.6 (7.1-21.1) | 0.018 |
|  | Albumin (g/L), n=34 | 31.5 (28.6-33.8) | 32.5 (28.9-34.7) | 0.371 |

Abbreviations: ALT, alanine transaminase; AST, aspartate transferase; CAZ-AVI, ceftazidime-avibactam; eGFR, estimated glomerular filtration rate.

**Supplementary Table 7 Comparison of safety between CAZ-AVI treatment containing and not containing tigecycline**

| **Treatment strategy** | **Laboratory variables** | **Before salvage treatment** | **After salvage treatment** | ***P*** |
| --- | --- | --- | --- | --- |
| **CAZ-AVI combination**  **not containing tigecycline** | Creatinine (umol/L), n=83 | 69.0 (42.0-125.0) | 56.0 (34.2-104.0) | 0.212 |
|  | eGFR, n=82 | 96.7 (48.9-122.7) | 104.6 (60.5-133.7) | 0.195 |
|  | ALT (U/L), n=83 | 31.0 (14.0-52.0) | 29.0 (14.0-55.0) | 0.910 |
|  | AST (U/L), n=83 | 38.0 (23.0-38.0) | 33.0 (22.0-63.0) | 0.582 |
|  | Total bilirubin (umol/L), n=83 | 14.3 (8.2-27.1) | 8.8 (6.1-14.0) | 0.001 |
|  | Albumin (g/L), n=83 | 29.5 (26.5-33.0) | 32.4 (29.5-35.1) | 0.001 |
| **CAZ-AVI combination**  **containing tigecycline** | Creatinine (umol/L), n=21 | 65.0 (38.0-144.5) | 52.0 (34.5-146.5) | 0.624 |
|  | eGFR, n=21 | 107.1 (39.2-132.2) | 116.7 (41.1-137.9) | 0.538 |
|  | ALT (U/L), n=21 | 27.0 (15.5-117.0) | 39.0 (27.0-99.5) | 0.580 |
|  | AST (U/L), n=21 | 50.0 (23.0-81.0) | 40.0 (29.0-87.0) | 0.940 |
|  | Total bilirubin (umol/L), n=21 | 18.3 (9.9-44.4) | 17.5 (10.0-43.2) | 0.960 |
|  | Albumin (g/L), n=21 | 29.7 (25.1-33.2) | 31.3 (28.4-34.0) | 0.352 |

Abbreviations: ALT, alanine transaminase; AST, aspartate transferase; CAZ-AVI, ceftazidime-avibactam; eGFR, estimated glomerular filtration rate.
